# Effect of Paternal Body Mass Index on Cumulative Live Birth Rates: Retrospective Analysis of 3048 Embryo Transfers in Couples Using Autologous Gametes

**DOI:** 10.3390/cells13221836

**Published:** 2024-11-06

**Authors:** Laura Mossetti, Irene Hervás-Herrero, María Gil-Juliá, Ana Navarro Gomez-Lechon, Rosa María Pacheco-Rendón, Rocío Rivera-Egea, Nicolás Garrido-Puchalt

**Affiliations:** 1 IVIRMA Global Research Alliance, IVIRMA Roma, Via Federico Calabresi, 11, 00169 Rome, Italy; 2 IVIRMA Global Research Alliance, IVI Foundation, Instituto de Investigación Sanitaria La Fe (IIS La Fe), Avenida Fernando Abril Martorell, 106-Torre A, Planta 1, 46026 Valencia, Spain; irene.hervas@ivirma.com (I.H.-H.); maria.gil@ivirma.com (M.G.-J.); ana.navarro@ivirma.com (A.N.G.-L.); rosa.pacheco@ivirma.com (R.M.P.-R.); 3 IVIRMA Global Research Alliance Andrology Laboratory and Sperm Bank, IVIRMA Valencia, Plaza de la Policia Local 3, 46015 Valencia, Spain; rocio.rivera@ivirma.com

**Keywords:** body mass index, ART, IVF, ICSI, clinical outcomes

## Abstract

Obesity is a multifactorial disease present worldwide and correlated with hormonal alterations that may cause a decrease in reproductive outcomes and seminal quality. However, the specific mechanisms involved are unknown. This led us to examine the relationship between paternal body mass index (BMI) and clinical reproductive outcomes by evaluating the cumulative live birth rates (CLBRs) per number of embryo transfers (ETs), embryos replaced (EmbRs), and oocytes used (OUs) in consecutive treatments until achieving the first newborn. A retrospective study was performed, and Kaplan–Meier survival curves were created to observe CLBRs with regard to the paternal BMI, adjusted by relevant confounders through Cox regression models. The participants were couples undergoing intracytoplasmic sperm injection (ICSI) and ET in Spanish IVIRMA clinics using autologous gametes. The cohort was subdivided based on paternal BMI: normal (18.5–24.99 kg/m^2^) (N), overweight (25–29.99 kg/m^2^) (OV), or obese (≥30 kg/m^2^) (OB) patients. A total of 4750 ICSI cycles were included, encompassing 49,485 mature oocytes, 23,963 blastocysts, and 3048 ETs. When calculating CLBRs based on the number of ETs carried out until live birth was achieved, no statistically significant differences were found (*p* = 0.72). After adjusting for maternal age and BMI, female infertility diagnosis, the use of preimplantation genetic testing, and the number of ETs, Cox regression showed that there were no statistically significant differences between the BMI groups (HR: 0.94 [95% CI: 0.7–1.2]; *p* = 0.59). When calculating CLBRs considering EmbRs, there was a similarity between the BMI groups (*p* = 0.57). However, there were no statistically significant differences in the adjusted Cox regression (HR: 0.93 [95% CI: 0.7–1.2]; *p* = 0.51). Finally, when calculating CLBRs considering OUs, the results were comparable among BMI subgroups (*p* = 0.75), and there were no statistically significant differences with adjusted Cox regression (HR: 0.95 [95% CI: 0.8–1.2]; *p* = 0.66). In conclusion, paternal BMI was not associated with clinical reproductive outcomes when considering the ETs, EmbRs, or OUs needed to reach the first liveborn (LB).

## 1. Introduction

Obesity is a metabolic disease determined by lifestyle as well as environmental and genetic factors [1] that has been classified since 1998 by the World Health Organization (WHO) as a global epidemic, with more than 650 million affected adults in the world.

According to a recent WHO statistics report, one in six adults is obese, and individuals living with overweight or obesity contribute to almost 2.8 million annual deaths worldwide (WHO, 2017). The negative consequences for conception are also increasing, with the increase in obesity in men of reproductive age having tripled since the 1970s [2].

Obesity is defined as an excessive accumulation of fat that can have consequences for health [3]. An individual is classified as overweight when their body mass index (BMI) is equal to or greater than 25 kg/m^2^, obese when it is equal to or greater than 30 kg/m^2^, and severely obese when it is equal to or greater than 40 kg/m^2^ [4].

The interest in investigating the consequences of obesity for male reproduction is rising. Recent clinical studies have demonstrated the negative impact of obesity on male reproductive function, implying that obesity is correlated with an increase in subfertility and infertility cases [5].

However, studies evaluating the effect of male obesity on normal physiological parameters of spermatozoa have reported inconclusive results. Individuals living with overweight or obesity can show reduced sperm motility and concentration, increased sperm DNA damage, lower embryo implantation rates, and acrosome reaction decline compared to normal-BMI patients [6]. In a 2014 study, increasing BMI was correlated with a decrease in seminal volume, and this result was confirmed by another study showing that not only is there a significantly negative association between sperm concentration and BMI but also that the proportion of morphologically normal sperm is inversely related to BMI [7].

With access to intracytoplasmic sperm injection (ICSI), reduced sperm quality does not necessarily reduce the possibility of conceiving a child, which is the main indicator of reproductive capacity. However, a recent meta-analysis demonstrated that elevated BMIs are correlated with a significant decrease in the pregnancy rate and live birth rate (LBR) following ICSI, concluding that BMI is a factor influencing the success of artificial reproductive technologies (ARTs) [8]. On the contrary, Arabipoor’s group reported no significant impact of increased paternal BMI on the LBR after ICSI and argued that the correlation only exists in overweight and obese women with lower LBRs [9].

The negative impact of elevated paternal BMI goes beyond embryonic development, affecting the clinical pregnancy rate [10,11], ongoing pregnancy, and LBRs [12]. Notably, this impact may be underestimated as reproductive clinical outcomes are often calculated per embryo transfer (ET), considering only the first and best-quality embryo transferred. This approach does not take into account the contributions of the remaining embryos in the cohort that can potentially contribute to reproductive success in subsequent transfers. Therefore, current assessments of paternal BMI influences on reproductive outcomes may be biased and/or incomplete when they only compare the best embryos between different cohorts and do not account for the additional reproductive probabilities from surplus frozen embryos [12].

Evidence on the impact of paternal BMI is scarce and contradictory, and there is a need to carry out studies to clarify this issue. The impacts of paternal BMI on clinical outcomes, sperm quality, and the incidence of diseases in offspring merit further investigation. Given that our group previously demonstrated that the cumulative live birth rate (CLBR) is a more accurate estimate of the probability of achieving a newborn based on male BMI [13,14], the main objective of this study was to evaluate the relationship of paternal BMI on ICSI outcomes, measuring success with the CLBR per ET, per embryo replaced (EmbR), and per number of mature oocytes necessary to achieve a live birth.

## 2. Materials and Methods

### 2.1. Study Population

This retrospective, observational, multicentric cohort study was performed using clinical data from patients undergoing ICSI cycles using autologous oocyte and sperm in Spanish IVIRMA fertility clinics (Alicante, Almeria, Barcelona, Bilbao, Burgos, Las Palmas, Madrid, Malaga, Mallorca, Murcia, Pamplona, Sevilla, Valencia, Vigo and Zaragoza) from January 2018 to December 2023. Baseline patient characteristics (i.e., age, height, weight, ethnicity, smoking habits, clinical history) and ICSI cycle outcomes were exported from internal medical records.

Couples with endometriosis, karyotype alteration, maternal age, low ovarian reserve, premature ovarian failure, polycystic ovarian syndrome, teratozoospermia, oligozoospermia, and unexplained infertility were included in this study. Cases in which sperm samples were obtained from testicular biopsy or epididymis aspiration were excluded. Cycles with incomplete study data were also excluded from analysis.

Participants were classified by paternal BMI: normal weight (18.5–24.99 kg/m^2^) (N), overweight (25–29.99 kg/m^2^) (OV), and obese (≥30 kg/m^2^) (OB). We excluded underweight patients due to the small number of men belonging to this group.

This study was approved by the Research Ethics Committee for the use of anonymized patient data (2104-FIVI-029-NG).

### 2.2. IVF Laboratory Procedures

Ejaculated sperm samples were collected in a sterile container after 3–5 days of sexual abstinence. Following 30 min of liquefaction, sperm concentration, volume, motility, and morphology were evaluated in a Makler counting chamber (Sefi Laboratories, Tel Aviv, Israel), according to the World Health Organization’s criteria (WHO 2010). The swim-up technique, density gradient centrifugation or washing were used to capacitate sperm for ICSI [15]. Fresh or frozen [16] mature (metaphase II; MII) oocytes were inseminated by ICSI [17] and cultured (37 °C, 5% CO_2_, 6% O_2_). Fertilization was confirmed X hours later, with the visualization of pronuclei and polar bodies. Embryos were cultured to the blastocyst stage (day 5) under a controlled environment and morphologically evaluated on day 3 and day 5 of culture using an inverted microscope at ×400 magnification. Blastocyst quality was evaluated according to the ASEBIR guidelines (2015), based on the inner cell mass and trophectoderm morphology. Good-quality embryos were selected for transfer or vitrification. When medically indicated for advanced maternal age, altered karyotype, implantation failure, male factors, or recurrent miscarriage, blastocysts were biopsied for preimplantation genetic testing (PGT). Lastly, one or two blastocysts were transferred (fresh or frozen) after hormone replacement therapy for endometrial preparation or a spontaneous ovulatory cycle [18], in accordance with national regulations and depending on the patients’ possibilities. While single ETs are routine clinical practice, couples with poor embryo quality, repeated failures, and advanced maternal age were eligible for double embryo transfer.

### 2.3. Clinical Data Collection

Clinical outcomes included the implantation rate (calculated based on the number of gestational sacs to embryo(s) transferred), biochemical pregnancy rate (calculated based on the cycles resulting in a serum beta human chorionic gonadotropin (*β*-hCG) > 10 IU/L measured 14–16 days post-ICSI), clinical pregnancy rate (calculated based on a positive *β*-hCG test 21–23 days post-ICSI or ultrasound-visualization of the fetal pole and heartbeat by 6.5–7 weeks of pregnancy), and ongoing pregnancy rate (calculated based on the ultrasound-visualization of intrauterine fetal development at week 12 of gestation). The biochemical miscarriage rate was defined as the absence of an intrauterine pregnancy after a positive *β*-hCG test result. The clinical miscarriage rate was defined as a spontaneous miscarriage before 12 weeks of gestation. All clinical rates were expressed per ET, except for implantation rates, which were expressed per EmbR.

The CLBR was determined as the proportion of deliveries with at least one live birth per ET, EmbRs, and per number oocyte needed to obtain a live birth, including all fresh and/or frozen cycles.

### 2.4. Statistical Analysis

Statistical analysis was conducted using R software (version 4.2.1; R Foundation for Statistical Computing, Vienna, Austria). Statistical significance was considered when *p* value < 0.05. The study cohort was subdivided by paternal BMI, which was considered a categorical variable whose odds ratios (OR) were obtained and expressed with their 95% CI. Clinical and demographic parameters are presented as means or proportions with their corresponding 95% confidence interval (95% CI) and were compared using Student’s *t* and Chi-square test, respectively. The cumulative probability of achieving a live birth was evaluated using the Kaplan–Meier estimator according to the total number of ETs, number of consecutive EmbRs, and number of OUs until a live birth was achieved [13,14]. All CLBR data are presented as the CLBR (95% CI). Survival rates of all fresh or frozen ETs, independent of whether they resulted in a live birth or not, were categorized by paternal BMI and compared using the Mantel–Cox test. To calculate the number of OUs until an LB, the oocytes that were inseminated and developed into transferred or nonviable blastocysts were included. Clinical outcomes that may impact the probability of an LB were analyzed considering the CLBR as the dependent variable and the number of ETs, EmbR, and OU as the independent variables. The covariates assessed were maternal age and BMI, infertility diagnosis, use of PGT, and number of ETs. 

## 3. Results

### 3.1. Baseline Patients and ART Characteristics

Our study included a total of 3935 patients who collectively underwent 4750 ICSI cycles using autologous oocytes. The cohort included 1829 N, 1655 OV, and 451 OB men. A total of 49,485 MII oocytes developed into 23,963 blastocysts. Of the 3048 ETs performed by the time this study was conducted, 1153 resulted in live births. A descriptive comparison of the paternal BMI-based subgroups is shown in Table 1. The overall mean paternal age of the cohort was 40.3 years (40.1–40.4), and the paternal BMI was 25.9 kg/m^2^ (25.8–25.9). Apart from maternal and paternal BMI, paternal age, oocyte state, and sperm capacitation method, there were no significant differences between normal and obese groups.

A subgroup analysis of the seminal parameters is presented in Table 2.

There were no significant differences between BMI subgroups in relation to seminal volume, sperm concentration, progressive motility of sperm, or total motile sperm count. However, the proportion of non-progressive sperm was significantly higher in patients with a normal BMI compared with patients living with obesity.

### 3.2. Paternal BMI and Clinical Outcomes

The clinical reproductive outcomes of ICSI/ET in paternal BMI subgroups are presented in Table 3. Our analysis showed no significant differences for implantation, biochemical pregnancy, clinical pregnancy, ongoing pregnancy, biochemical miscarriage, and clinical miscarriage rates per ET between the BMI subgroups. Adjusting for confounding factors, such as maternal infertility diagnosis, maternal age and BMI, the use of PGT, and the number of ETs, Cox regression showed there were no statistically significant differences in the odds ratios between paternal BMI subgroups (Table 4).

### 3.3. CLBR According to ET

**Figure 1:**
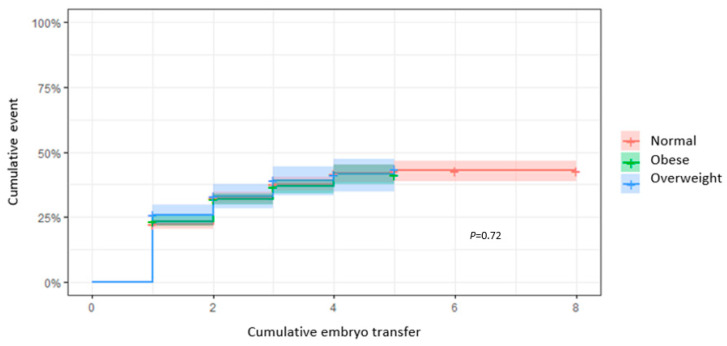
CLBRs in relation to the total number of ETs. Kaplan–Meier curves plotting hazard functions for the CLBR according to the number of ETs until a live birth was achieved.

When calculating the CLBR in relation to the number of ETs performed until the first live birth was obtained, there were no statistically significant differences observed between paternal BMI subgroups (*p* = 0.72) (Figure 1). After the first ET, the CLBR was 31.1% (95% CI: 28.5–33.6) for men with a normal BMI, 27.7% (95% CI: 25.2–30.2) for men living with overweight, and 32.1% (95% CI: 26.6–37.2) for men living with obesity. Considering three ETs, the CLBRs were, respectively, 50.0% (95% CI: 45.4–53.3), 42.6% (95% CI: 38.8–46.2), and 46.6% (95% CI: 38.1–53.9), while after four ETs, the CLBRs rose to 52.9% (95% CI: 47.3–57.9), 49.1% (95% CI: 42.4–55.0), and 49.6% (95% CI: 39.3–58.1). Cox modelling confirmed a non-significant correlation between paternal BMI and the CLBR (HR: 0.94 [95% CI: 0.7–1.2]; *p* = 0.59).

### 3.4. CLBR According to the EmbR

**Figure 2:**
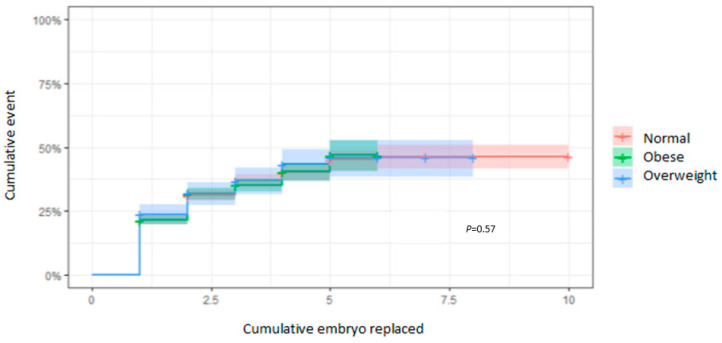
CLBRs in relation to the total of EmbR. Kaplan–Meier curves plotting hazard functions for the CLBR depending on the number of EmbRs required to achieve a live birth.

After one EmbR, the CLBR was 29.8% (95% CI: 27.2–32.3) for men with a normal BMI, 25.6% (95% CI: 23.2–28.0) for men living with overweight, and 29.4% (95% CI: 24.0–34.4) for men living with obesity. After three EmbRs, the CLBRs, respectively, increased to 49.2% (95% CI: 45.3–52.8), 40.4% (95% CI: 37.0–43.7), and 44.9% (95% CI: 37.3–51.7); however, there were no significant differences between groups (*p* = 0.57) (Figure 2). These observations were confirmed by the comparable results obtained with the adjusted Cox regression (HR: 0.93 [95% CI: 0.7–1.2]; *p* = 0.51).

### 3.5. CLBR According to the Number of MII Oocytes Used

**Figure 3:**
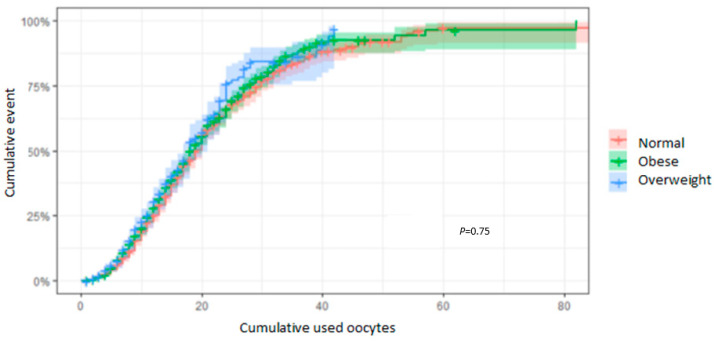
CLBRs in relation to the total number of OUs. Kaplan–Meier curves plotting the hazard functions for the CLBR depending on the number of MII oocytes required to achieve a live birth.

The CLBR according to the number of OUs was not significantly different between paternal BMI subgroups (*p* = 0.75) (Figure 3). For example, in couples with eight OUs, the CLBR was 13.7% (95% CI: 11.4–16.1) for men with a normal BMI, 15.3% (95% CI: 12.8–17.8) for men living with overweight, and 14.8% (95% CI: 9.8–19.6) for men living with obesity. After 12 OUs, the CLBRs rose to 30.3% (95% CI: 26.9–33.6), 29.5% (95% CI: 26.1–32.8), and 32.1% (95% CI: 24.9–38.5), respectively.

When using 16 oocytes, the CLBRs were, respectively, 48.1% (95% CI: 44.0–51.9), 44.2% (95% CI: 40.0–48.0), and 44.5% (95% CI: 36.2–51.7) for the N, OV, and OB subgroups, with no statistically significant differences between them. Moreover, the Cox regression model also confirmed this result (HR: 0.95 [95% CI: 0.8–1.2]; *p* = 0.66) (Figure 3).

## 4. Conclusions

The first reports of the impact of male obesity on infertility were published less than a decade ago [13], but the extent of this relationship and its mechanisms remain controversial.

In this study, we evaluated whether paternal BMI was associated with seminal parameters and clinical outcomes in couples undergoing ICSI and ET. We observed that men living with obesity had the same sperm concentration, mobility, and morphology as men with a normal BMI. These findings contrast those of Jensen et al. [19], who found a higher incidence of oligozoospermia in overweight and obese men compared to men with normal weight (24.4% vs. 21.7%, respectively). However, aligning with our findings, they did not observe a correlation between elevated paternal BMI and the percentage of motile sperm. In another study, the sperm concentration was found to be significantly lower in men living with obesity than those without. Further, the sperm concentration in the obese group was found to continuously decrease with advancing paternal age [20]. Kort et al. claimed that male BMI was negatively correlated with the total number of normal spermatozoa, observing that men with a BMI greater than 25 kg/m^2^ presented fewer normal chromatin-intact motile sperm per ejaculate [21]. Elevated paternal BMI might alter hormonal profiles, resulting in defective spermatogenesis, reduced semen quality [22,23], and compromised blastocyst development [24]. Although some studies found no association between paternal BMI and the percentage of motile sperm, others have shown BMI to be negatively correlated with the motile sperm count. This may be due to variable classifications of BMI subgroups between studies. In our study, we found an impact on non-progressive sperm only in men who were categorized as obese (BMI ≥ 30 kg/m^2^).

Our study highlighted that paternal BMI was not correlated with clinical outcomes in couples undergoing ICSI. Indeed, adjusting for confounding factors, men considered to be obese had the same reproductive outcomes as normal-weight men. Although several groups have explored the impact of male BMI on clinical outcomes during IVF, they reported contradictory conclusions. A study of infertile couples concluded that obese men had a lower chance of achieving a clinical pregnancy than men of normal weight. However, there was no statistical difference for the early miscarriage rate or ectopic pregnancy rate between normal and obese groups [25]. Alternatively, another study associated elevated paternal BMI with a lower clinical pregnancy rate and found that the odds of a live birth in couples with obese males was 84% lower than in couples where the male had a normal BMI. Notably, this study found no significant correlation between paternal BMI and fertilization or pregnancy rates [26]. Bakos et al. (2011) also reported that there was no association of male BMI with the overall fertilization rate. However, there was a statistically significantly negative correlation between paternal BMI and pregnancy or live birth rates [26]. Another retrospective study of patients undergoing ARTs demonstrated that paternal obesity was correlated with lower clinical pregnancy rates compared with normal male BMI. Nevertheless, the authors suggested that ICSI may overcome obesity-related impairments in the sperm–oocyte interaction [24].

In ART, achieving a live birth is the goal, but there is limited information on how men living with overweight can achieve this outcome. Thus, we explored the potential effects of male BMI on the CLBR. In this retrospective study, we concluded that paternal BMI does not reduce the CLBR nor individual clinical reproductive outcomes when evaluating them per ET, EmbR, or OU. Disproving our hypothesis, we found that overweight or obese fathers carried no additional risks for the CLBR. To our knowledge, this is the first study evaluating the impact of paternal BMI on CLBRs, and this approach provides a more reliable assessment of the relationship of paternal BMI on IVF cycle success with autologous gametes.

The main limitations of our study include its retrospective design, which limits the ability to control and collect key parental factors that may influence the results. Furthermore, the lack of patient follow-up in some cases may bias outcomes and limit the awareness of adverse events that may have occurred, which could lead to an underestimate of the associated risks. These clinical factors are not registered in the patient’s medical record by physicians before starting ART (e.g., smoking, time to pregnancy, history of pregnancy loss, and comorbid medical condition) or information not provided by couples in subsequent pregnancies. Despite the statistical analyses being adjusted to account for confounding factors in our large sample size, future randomized controlled trials would be helpful to confirm the results presented herein.

In conclusion, our study revealed that paternal BMI ≥ 25 kg/m^2^ has no negative association with clinical reproductive outcomes following ICSI using autologous sperm and oocytes. However, we emphasize that overweight and obese men seeking ARTs should still be counselled to lose weight to improve their general health and economic costs of treating associated comorbidities. Lifestyle interventions and holistic treatment of couples undergoing ART can help improve clinical management and IVF outcomes.

## Figures and Tables

**Table 1:** Baseline patient characteristics and IVF cycle parameters according to paternal BMI at conception.

	N (*n* = 1829)		OV (*n* = 1655)		OB (*n* = 451)		*p* Value
	Mean or Proportion	95% CI	Mean or Proportion	95% CI	Mean or Proportion	95% CI	
Paternal age (years)	40.0	39.8–40.2	40.6	40.4–40.9	40.4	39.9–40.8	<0.001
Paternal BMI (kg/m^2^)	23.2	23.1–23.3	27.0	26.9–27.1	32.6	32.4–32.8	<0.001
Maternal age (years)	38.2	38.1–38.4	38.3	38.1–38.5	38.0	37.6–38.4	0.31
Maternal BMI (kg/m^2^)	22.2	22.0–22.3	23.4	23.3–23.6	25.5	25.1–25.9	<0.001
Female indications							0.19
Altered karyotype	0.4% (9/2112)	0.2–0.8	0.4% (8/1984)	0.2–0.8	0.8% (4/515)	0.2–2.1	
Low ovarian reserve	10.9% (230/2112)	9.6–12.3	10.1% (200/1984)	8.8–11.5	7.8% (40/515)	5.7–10.5	
Endometriosis	1.9% (40/2112)	1.4–2.6	1.2% (24/1984)	0.8–1.8	2.5% (13/515)	1.4–4.4	
Premature ovarian failure	0.1% (3/2112)	0.04–0.4	0.05% (1/1984)	0–0.3	0% (0/515)	0–0.9	
Polycystic ovary syndrome	2.7% (58/2112)	2.1–3.6	2.4% (47/1984)	1.8–3.2	2.9% (15/515)	1.7–4.9	
Other factors	83.9% (1772/2112)	82.2–85.4	85.9% (1704/1984)	84.3–87.4	86.0% (443/515)	82.6–88.8	
Number of MII oocytes	8.3	8.1–8.4	8.3	8.0–8.5	8.3	8.0–8.5	0.89
Oocyte state							0.06
Fresh	80.5% (1758/2185)	80.1–80.7	80% (1624/2030)	78.5–82.5	85.6% (458/535)	85.1–86.1	
Vitrified	2.2% (48/2185)	2.1–2.3	2.2% (44/2030)	2.1–2.3	1.7% (9/535)	1.5–1.9	
Mixed	17.3% (379/2185)	16.9–17.6	17.8% (362/2030)	17.6–17.9	12.7% (68/535)	12.1–13.2	
Cycle type							0.02
Stimulated	94.5% (2064/2185)	94.1–94.8	92.1% (1870/2030)	91.5–92.6	94.8% (507/535)	94.4–95.2	
Natural	1.1% (24/2185)	1.0–1.2	1.3% (27/2030)	1.0–1.5	1.2% (4/535)	1.0–1.4	
Substituted	4.4% (97/2185)	4.1–4.6	6.5% (133/2030)	6.2–6.9	4.5% (24/535)	4.1–4.9	
Capacitation method							0.02
Density gradient	68.7%	68.1–68.9	66.2%	66.1–66.3	62.1%	61.8–62.4	
Swim-up	26.5%	26.1–26.8	28.7%	28.2–29.1	33.6%	33.4–33.9	
Only washed	4.7%	4.1–4.9	5.1%	4.7–5.3	4.2%	3.9–4.4	
ET with PGT-A							0.08
Yes	14.6% (139/952)	14.3–14.9	18.4% (175/953)	18.1–18.6	17.2% (39/227)	17.1–17.3	
No	85.4% (813/952)	85.1–85.6	81.6% (778/953)	81.3–81.9	82.8% (188/227)	82.4–83.2	

Data are expressed as a proportion for categorical variables or mean for continuous variables with corresponding 95% confidence intervals (CI). The *p* value was determined by the Student’s *t*-test.

**Table 2:** Comparison of seminal parameters based on paternal BMI at conception.

	N (*n* = 1829)		OV (*n* = 1655)		OB (*n* = 451)		
	Mean	95% CI	Mean	95% CI	Mean	95% CI	*p* Value
Seminal volume (mL)	2.8	2.7–2.8	2.8	2.7–2.8	2.7	2.6–2.8	0.81
Sperm concentration (×10^6^/mL)	43.8	42.3–45.2	41.8	40.3–43.3	41.5	38.5–44.5	0.14
Progressive sperm (%)	39.2	38.5–39.9	39.3	38.5–40.1	40.4	38.8–41.9	0.42
Non-progressive sperm (%)	9.5	9.2–9.8	9.4	9.0–9.7	8.6	7.9–9.2	0.04
Total motile sperm count	118.0	108.0–128.1	112.8	101.6–124.1	115.1	92.3–137.9	0.79

Data are presented as a mean (*n*) with the corresponding 95% confidence interval (95% CI). *p* values were determined by the Student’s *t*-test.

**Table 3:** Clinical outcomes per ET according to BMI subgroups.

	N		OV		OB		*p* Value
	Proportion (*n* = 1829)	95% CI	Proportion (*n* = 1655)	95% CI	Proportion (*n* = 451)	95% CI	
Implantation rate per EmbR	57.6% (723/1255)	54.8–60.4	54.6% (687/1258)	51.8–57.4	58.5% (172/294)	52.6–64.2	0.23
Biochemical pregnancy rate per ET	61.6% (842/1367)	58.9–64.2	58.6% (801/1367)	55.9–61.2	62.1% (195/314)	56.5–67.4	0.22
Clinical pregnancy rate per ET	52.3% (715/1367)	49.6–54.9	49.8% (681/1367)	47.1–52.5	53.8% (169/314)	48.1–59.4	0.28
Ongoing pregnancy rate per ET	42.6% (583/1367)	40.0–45.3	39.5% (540/1367)	36.9–42.2	43.3% (136/314)	37.8–49.0	0.18
Biochemical miscarriage rate per ET	8.7% (119/1367)	7.3–10.4	8.3% (113/1367)	6.9–9.9	7.6% (24/314)	5.1–11.3	0.81
Clinical miscarriage rate per ET	7.9% (109/1367)	6.6–9.6	8.3% (114/1367)	6.9–9.9	7.9% (25/314)	5.3–11.7	0.93

Data are presented as a mean (*n*) with the corresponding 95% confidence interval (95% CI). *p* values were determined by the Student’s *t*-test.

**Table 4:** Odds ratios per embryo transfer according to paternal BMI groups, adjusted by maternal age and BMI, female infertility diagnosis, the use of PGT, and the number of ETs.

	OR	95% CI	*p* Value
Implantation rate per EmbR	1.0	0.9–1.1	0.79
Biochemical pregnancy rate per ET	0.9	0.9–1.0	0.82
Clinical pregnancy rate per ET	1.0	0.9–1.1	0.79
Ongoing pregnancy rate per ET	1.0	0.9–1.0	0.1
Biochemical miscarriage rate per ET	0.9	0.9–1.0	0.3
Clinical miscarriage rate per ET	1.0	0.9–1.0	0.9

*p* values were determined by the Student’s *t*-test.

## Data Availability

All data supporting the findings of this study are available within the paper. The data presented in this study are openly available at https://pubmed.ncbi.nlm.nih.gov/, all accessed starting 1 January 2023.
